# A Reinforcement Learning Framework to Discover Natural Flavor Molecules

**DOI:** 10.3390/foods12061147

**Published:** 2023-03-08

**Authors:** Luana P. Queiroz, Carine M. Rebello, Erbet A. Costa, Vinícius V. Santana, Bruno C. L. Rodrigues, Alírio E. Rodrigues, Ana M. Ribeiro, Idelfonso B. R. Nogueira

**Affiliations:** 1 LSRE-LCM—Laboratory of Separation and Reaction Engineering-Laboratory of Catalysis and Materials, Faculty of Engineering, University of Porto, Rua Dr. Roberto Frias, 4200-465 Porto, Portugal; 2 ALiCE—Associate Laboratory in Chemical Engineering, Faculty of Engineering, University of Porto, Rua Dr. Roberto Frias, 4200-465 Porto, Portugal; 3 Chemical Engineering Department, Polytechnic School Federal University of Bahia, Salvador 40210-630, Brazil; 4 Chemical Engineering Department, Norwegian University of Science and Technology, Sem Sælandsvei 4, Kjemiblokk 5, N-7491 Trondheim, Norway

**Keywords:** scientific machine learning, deep generative model, deep reinforcement learning, flavor engineering

## Abstract

Flavor is the focal point in the flavor industry, which follows social tendencies and behaviors. The research and development of new flavoring agents and molecules are essential in this field. However, the development of natural flavors plays a critical role in modern society. Considering this, the present work proposes a novel framework based on scientific machine learning to undertake an emerging problem in flavor engineering and industry. It proposes a combining system composed of generative and reinforcement learning models. Therefore, this work brings an innovative methodology to design new flavor molecules. The molecules were evaluated regarding synthetic accessibility, the number of atoms, and the likeness to a natural or pseudo-natural product. This work brings as contributions the implementation of a web scraper code to sample a flavors database and the integration of two scientific machine learning techniques in a complex system as a framework. The implementation of the complex system instead of the generative model by itself obtained 10% more molecules within the optimal results. The designed molecules obtained as an output of the reinforcement learning model’s generation were assessed regarding their existence or not in the market and whether they are already used in the flavor industry or not. Thus, we corroborated the potentiality of the framework presented for the search of molecules to be used in the development of flavor-based products.

## 1. Introduction

The sensation of a flavor is defined by the Encyclopaedia Britannica as the attribute of a substance perceived within the mouth produced by the senses of smell, taste, and touch [1]. From an anatomical standpoint, this sensation is distinguished by the taste buds in the oral cavity, in which is the pharynx, palate, larynx, and tongue. An adult has approximately 10,000 taste buds. The flavor response in the taste buds happens by identifying chemicals present in food and beverages, for example, and their translation into nerve signals. Understanding the biological flavor process and response is pivotal in developing the food, beverage, and flavor industries [2].

Flavor plays an essential role in several products found in modern markets. Furthermore, new flavors are required by manufacturers for product development and innovation. In this context, researching new flavors and developing innovative production technologies is constantly undertaken. However, society is observing increasing consumption of processed products and improved fast-food products. These products are composed of several food additives and flavoring agents. More recently, expanding awareness of the importance of a healthy lifestyle has provoked a rise in the search for foods labeled with a natural flavoring agent. This movement has motivated the growth of flavors’ R&D activities and investments in new flavor-based products [3]. In 2020, the flavors’ and fragrances’ market size from food and beverage products was appraised at EUR 26.53 billion, and the prospect is that by 2026, it will reach EUR 35.52 billion [4].

Although synthetic flavors are profitable and applied in food products, the heaviest impact on the market share is from natural flavors. This is a consequence of the intensified search for a healthy lifestyle and the increasing awareness of the hazardous effects of some synthetic flavors. The increase of this new lifestyle has also impacted the Beverage sector, which is continually growing and significantly influences the market. Overall, the largest end-use industries of flavors are beverages, bakery, savories and snacks, dairy and frozen products, confectionery, and pet food [5]. Such sectors are forecast to grow through increasing application and development of new products based on natural flavors [3]. However, other industries are also investing in flavoring their products to satisfy the consumers or make their products more appealing, such as producing flavored toothpaste.

According to the Regulation (EC) No. 1334/2008 of the European Parliament and of the Council of 16 December 2008 on flavorings and certain food ingredients with flavoring properties for use in and on foods and amending Council Regulation (EEC) No. 1601/91, Regulations (EC) No. 2232/96 and (EC) No. 110/2008 and Directive 2000/13/EC [6], the following is put forth:

- There is no distinction between nature-identical and artificial flavoring substances; both are referred to as “Flavoring substances”;
- The labeling of product’s ingredients as “natural flavoring substances” can only be applied exclusively for natural flavoring substances;
- Flavoring substances are defined as chemical substances obtained from chemical synthesis or isolated through chemical processes and natural flavoring substances;
- “Natural flavoring substance” is a flavoring substance produced by appropriate physical, enzymatic, or microbiological processes from a material of vegetable, animal, or microbiological origin.

The replication of natural flavor chemicals by synthetic molecules empowers the design of more stable, purer, potent, and cost-effective synthetic flavors. The discovery and combining of the molecules to express the multisensorial complexity as a nerve signal inherent to flavors is a trial-and-error process [7]. Additionally, the law and regulations must be taken into consideration when developing flavor-based products, especially concerning the environmental impact that the synthetic chemicals process can cause and the possible pathological state [8]. On that account, flavor engineering development is a high-cost and time-consuming process. In this way, the implementation of innovative technologies can be resourceful in reducing overheads and increasing efficiency. Consequently, scientific machine learning (SciML) puts forward a novel approach to this industry.

Machine learning (ML) tools are driving advances in the scientific field, especially the scientific machine learning adaptation, which is an emergent area in addressing specific domain challenges. SciML is a resource-saving and efficient method in general data mining, modular design, image processing, bioinformatics, game playing, and computational chemistry [9]. The implementation of machine learning in chemistry engineering is optimistic in designing, synthesizing, and generating molecules and materials [10]. Particularly, a modest emerging but promising tendency in implementing SciML tools is perceived in flavor engineering.

Leong et al. (2021) [11] applied machine learning to analyze and quantify the flavor in a matrix. The authors developed a surface-enhanced Raman scattering (SERS) taster that achieved outstanding accuracy in quantifying wine flavors using four receptors of the spectroscopic profiles. Impressive results were obtained responding to the struggles of the chemical analysis of flavor compounds. This made it possible to analyze the detection in a rapid and sensitive procedure. Bi et al. (2020) [7] also applied ML to improve other aspects of flavor engineering. In the mentioned case, an approach was developed to predict olfactometry results from gas chromatography–mass spectrometry/olfactometry (GC-MS/O). The olfactometry results can be achieved in 30 s with the proposed technology. However, the machine learning model presented an overfitting problem. The authors concluded that the technology could be improved even though it already has potential for olfactometry evaluation.

Regarding the two SciML techniques that are the focus of this work, i.e., the generative and reinforcement learning models, a brief assessment of the literature is required. For the generative model, the one implemented in the integrated system used in this work is based on the work of Mercado et al. (2021) [12]. Numerous approaches could have been chosen. However, based on the analysis of generative models presented by Zhang et al. (2021) [13], the GraphINVENT method presented a better performance when trained with the GDB-13 database of drug-like compounds. In their work, the deep molecular generative models analyzed were CharRNN, REINVENT, AAE, VAE, ORGAN, LatentGAN, and GraphINVENT. It is important to notice that deep generative models are applied for the design of drug molecules. In the literature, it is not possible to find records of these methods being applied for flavor engineering.

Reinforcement learning (RL) was introduced by Richard Sutton and Andrew Barto in 1984 based on behavioral psychology and is the third machine learning paradigm, along with supervised and unsupervised learning. RL’s application consists of an agent interacting and learning from an environment. Based on the experience amassed, the agent optimizes the defined objectives as cumulative rewards that must be maximized. The agent does not need to have all the information regarding the environment. It only requires the ability to interact with the environment and learn from it over time. This agent–environment relationship is based on value function, reward, policy, and model. Recently, the efficiency of RL in solving sequential decision-making problems has increased its popularity [8,14].

The sequential function of the RL model starts with the agent receiving an initial state based on the observation of the environment. At a time, the agent must take action. Each action leads to a consequence that can be a reward, state change, and observation. The information gathered from its actions’ results makes it possible for the agent to learn its purpose over time [8]. 

Artificial intelligence (AI) development for video games has been a largely studied field aiming to achieve human-level performances when testing video games [9,10,15]. The complex interactions between the agent and the virtual environment involve a decision-making problem. In this instance, the RL model presents itself as a feasible solution. Solving game challenges with RL has shown excellent results, alongside its combination with deep learning to improve the generalization and scalability of the model [15].

In the context of molecular design, it is possible to find in the literature numerous works that implemented the reinforcement learning technique, alongside generative models and/or convolutional neural networks, in order to design drug molecules. Once again, it is not possible to find in the literature records of these methods being applied for flavor engineering. For example, the work of Olivecrona et al. (2017) [16] proposed a system combining recurrent neural networks (RNN) and reinforcement learning to design drug-like molecules similar to the drug Celecoxib. The training results were considered successful; however, it was stated that target activity, the DMPK profile, and synthetic accessibility score must be considered in the reinforcement learning as a future work. In the same context, Born et al. (2021) [17] applied reinforcement learning to design anti-cancer molecules considering drug-likeness, synthesizability, and solubility. The PaccMann framework presented as results compounds that can be considered for the anti-cancer application with high predicted capacity. 

Although SciML applications can be found in the flavor engineering field, the whole potential of the technique is not yet deployed. The implementation of SciML in identifying and designing new flavoring molecules has not yet been explored in the field. Consequently, the SciML is a simple and reliable way to approach these challenges and produce new chemical molecules that can be synthesized. These goals were tackled in the context of this work within the deep reinforcement learning model. More specifically, it is not possible to find in the literature record of application of SciML in order to find flavor-like molecules that can be considered natural or be extracted from natural sources. It is indispensable to highlight that natural flavor molecules are discovered and obtained from plants, animals, or microorganisms through processes such as extraction, distillation, enzymolysis, roasting, and heating. The novel discoveries in this field are all obtained through trial-and-error processes and further detailed studies of natural sources according to the literature.

Hence, this work proposes a novel framework combining generative neural network models and reinforcement learning to develop new flavors and flavor-based products. This allows the possibility of generating new flavor molecules that can be synthesized and applied in industry to provide products that can be considered natural. Thus, addressing a critical challenge found in the modern flavor industry and pioneering the implementation of scientific machine learning to molecules’ design in flavor-based product development is of great importance.

## 2. Materials and Methods 

### 2.1. Background

**Figure 1:**
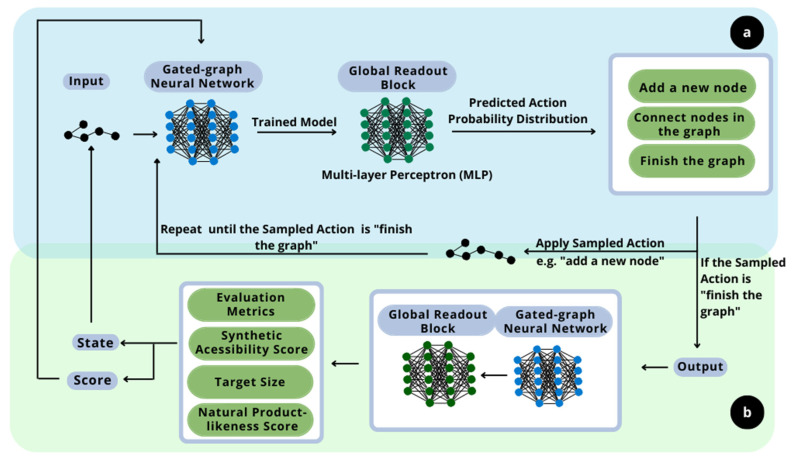
Overall methodology’s scheme, composed by generative model (**a**) and reinforcement learning model (**b**).

The principle of this work is the development of new molecules with specific sought-after properties and flavors. In this context, the proposed methodology consists of implementing two distinct deep learning techniques. The overall scheme is presented in Figure 1.

The generative model, the first deep learning technique implemented, as shown in section a in Figure 1, is based on the work of Queiroz et al. (2022) [18]. The deep generative model is a resourceful technique to recognize patterns of likelihood between samples. It learns how to construct and deconstruct molecules following a canonical deconstruction path in the numerous hidden layers of neural networks. The aim of the layers is to design new molecules with similar properties and structure. Those are calculated based on the resulting graphs of the current step and epoch and are given as output. They are also important to evaluate the impact of the randomly sampled choices. This ability to learn the probabilistic distribution regarding the molecules is the main reason for the implementation of this technique, as it assures that the designed molecules follow the structure of the flavor-based molecules given as input. Furthermore, it is important to highlight that the generative model implemented in a reinforcement learning context for flavor-based product development is a novelty approach. It was not possible to find in the literature any proposition of this methodology in the flavor engineering field, so it establishes this methodology as a contribution of this work.

The second step of the methodology is the implementation of the reinforcement learning technique, as shown in section b of Figure 1, alongside the deep neural network model of the generative model, to maximize the flavor-likeness of the molecule while carrying other desired properties [19]. This maximization is due to the application of rewards within the evaluation metrics, so the deep neural network improves the design of the molecules regarding the properties and characteristics of interest. Hence, a deep reinforcement learning process is proposed.

It is fundamental to highlight the significance of implementing scientific machine learning techniques as innovative as deep generative models and deep reinforcement learning to strengthen product development in a traditional and trial-and-error-based field such as flavor engineering. Similar premises have been put forward as novel frameworks in the pharmaceutical industry and bioinformatics fields. However, nothing close to the proposed work has ever been produced or presented in flavor engineering. 

### 2.2. Database

The first step of the methodology is the sampling of the database. In the development of this work, a web scraper code extracted the database from the website FlavorDB [20]. For this purpose, 921 valid molecules were retrieved along with chemical properties information and the flavor descriptors associated with the corresponding molecules. The properties related to the database are PubChem ID, chemical name, flavor descriptors of the molecule, and SMILES representation. A data curation step was performed automatically in all the databases to guarantee that all the SMILES representations were in canonical form. In sum, 417 flavor descriptors were identified, in which the descriptors are correlated to each other. 

**Figure 2:**
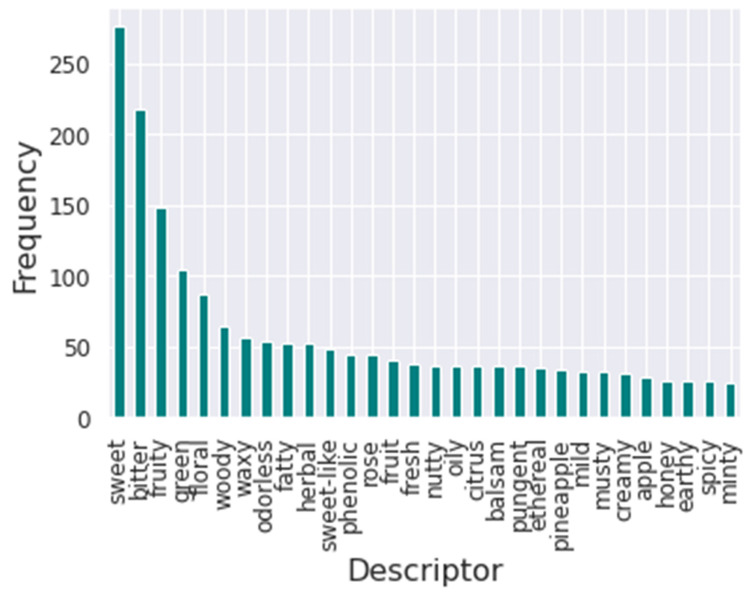
Bar plot of the 30 most common flavor descriptors’ frequency in the database.

Furthermore, the flavors’ frequency was analyzed to understand the impact of each flavor descriptor in training. Figure 2 presents the bar plot of the frequency of the thirty most common flavor descriptors in the database. It is possible to visualize that the most common descriptor is sweet, followed by bitter. Meanwhile, the least common descriptor in the database is winey.

**Figure 3:**
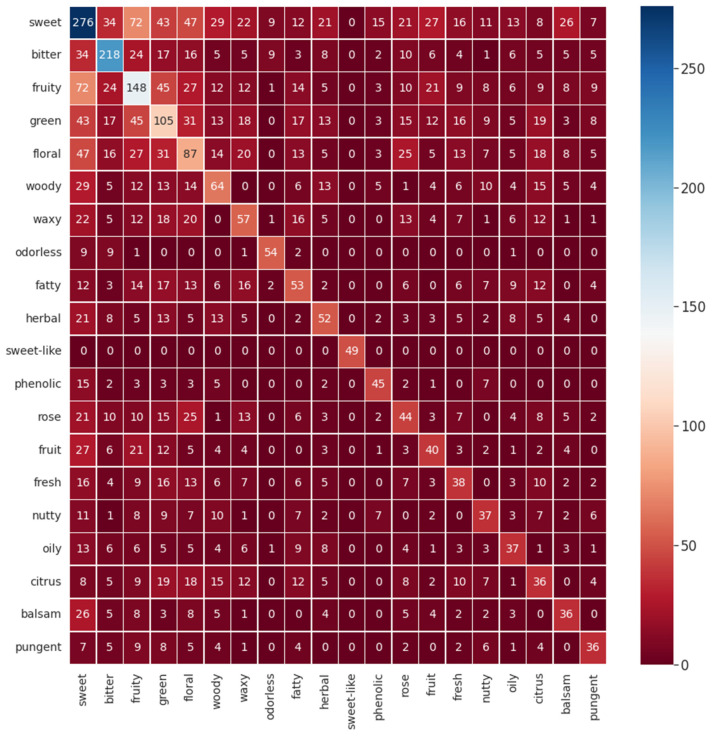
Co-occurrence heat map for the 20 most common flavor descriptors in the database.

To comprehend the possible co-occurrence of flavor descriptors for a single flavor molecule, a heatmap for the twenty most common descriptors in the database was made and is given in Figure 3. As expected, it is possible to visualize that, for example, sweet correlates with the descriptors green, floral, and fruity. Some correlations can be expected considering common sense and empirical experiences that all humans have throughout life. Correlating a piece of fruit with a sweet flavor is an ordinary situation as well as thinking that a rose flavor resembles a floral one, for example. However, other correlations are not so obvious, such as the correlation between citrus and woody. For this instance, this perception of flavor’s correlation is a significant resource for flavor engineering. 

One key step before training the neural networks is dividing the database into training, test, and validation sets. This division must always be made to subdue the overfitting in the model and to guarantee the model’s accuracy. In this work, 60% of the database was used for the training set, 20% for the test set, and 20% for the validation set.

### 2.3. Methodology

After the database was acquired, and the three sets were divided, each molecule of the database, one after another, was given as input to the deep generative model in the SMILES form. First, the molecule was preprocessed, so the model learned how to construct and deconstruct it following the canonical deconstruction path. Then, the obtained information followed the network path through the gated graph neural network (GGNN), introduced by Li et al. (2016) [21]. The resultant molecules were evaluated through evaluation metrics in intervals of epochs. The focus of this work is the implementation of reinforcement learning associated with the generative model, so the structure and methodology of the deep generative model will not be described. 

After the generative model is trained, the trained models are saved. The best-trained model in the generative neural network is the one used to train the proposed deep reinforcement framework. The database used in training the generative model is again given as input. The system of GGNN and global readout block is kept in the same manner as the prior architecture. However, the agent impacts the way the decisions are made to build a molecule. 

The possible actions are the same: adding a new node, connecting nodes in the graph, or finishing the graph. Simultaneously, the agent influences the choice of action and can influence the choice of atom type used in the valid molecule’s design [22]. The environment states are the set of all the chemical structures designed during the generation process. In other words, the environment is the generative model, and the states are the output of the generation process for the whole database. Deep reinforcement learning takes the batch of generated molecules as input for the agent and not each molecule, as the generative model does. It is essential to specify that, regardless of the reinforcement’s input, the generative model as the environment receives each molecule at a time.

To achieve better results, i.e., maximize the rewards, the weights in the CNN policy are updated according to the performance of the model. CNN’s weights also impact the results of the loss function. In these circumstances, the agent influence is in the weights in the calculation behind the generative model [22].

In a more straightforward perspective, compared to a video game, the environment is the deep generative model, the main character is the loss function’s weight, and the state is the output of the deep generative model.

The generated molecules are analyzed and receive a reward from the agent. These molecules are, once again, deconstructed and reconstructed but this time in the agent’s GGNN model. The mentioned process takes place to calculate the action probability distribution (APD) and its influence on the loss function in the same way as performed in the generative part. The APD is calculated through a SoftMax function [23]. The result obtained by the agent for the current RL batch is compared to the one obtained in the previous batch. 

The new action determined is applied to all the generated molecules aiming to estimate the success of this action. In other words, it predicts the number of molecules that will be fully terminated and valid when the generative model is reset with the updated weights. In this instance, the action that performs better is the one with the heaviest weight in the loss function. 

The designed molecules given as outputs of the generative model, which are fully finished and valid, are separated from the rest of the batch into a group of finished molecules. These molecules are given as an output of the agent for the corresponding batch. One relevant aspect of this segmentation is that the RL’s model considers the uniformity-completeness Jensen–Shannon divergence (UC-JSD) metric as the generation metric. For this instance, the generation’s success is also evaluated based on this specific metric. 

Another critical part of the “traditional” reinforcement learning structure used in games is the reward. The deep reinforcement learning proposed here also makes use of rewards. The definition of the rewards is essential for guiding the agent’s influence on the system and to enforce the system to follow the properties of interest in designing molecules. In this work, the final score comprises the synthetic accessibility score (SAScore) rewards, the target size, and the natural product-likeness score (NPScore). 

Ertl et al. (2009) [24] proposed the synthetic accessibility score. The score is calculated considering the contribution of the molecule complexity and the fragmented contributions. It is based on previous studies of a million known chemicals that were already synthesized. The molecule complexity score notices the possible presence of non-standard structural features. The final SAScore for each molecule ranges from one to ten. A SAScore of one means that the molecule is easily synthesized, and ten means that the molecule is exceedingly difficult to synthesize. The reward was defined so that the SAScore value is inversely proportional to the reward given. 

The natural product-likeness score was introduced by Ertl et al. (2008) [25]. The score is a Bayesian measure to quantify the similarity of a compound with a natural compound based on the analysis of its structural fragment. The reference source for the natural compounds is the CRC Dictionary of Natural Products (DNP). The score ranges between −5 and 5; the higher the score, the more like a natural product the molecule analyzed is. Around the value zero are the pseudo-natural products [26]. The term was proposed by Karageorgis et al. (2020) [27] and is applied to small molecules derived through the combination of natural products’ fragments.

The target size is defined as follows: if the number of nodes in the molecule’s graph is between zero and the predefined maximum, the reward is one, and otherwise, it is zero. The final score is the sum of the rewards for each RL batch, in which each contribution in the score has equal weight. However, the final score calculation does not consider the contribution of molecules that are not unique, invalid, or fully finished. 

It is relevant to notice that the rewards are important to “teach” the neural network and guide it to design molecules with the specific characteristics and properties to be applied in the industry. However, the molecular structure and its likelihood to a flavor molecule is not ensured by the reinforcement learning technique but by the generative model as it learns the probabilistic distribution related to the molecules.

The agent’s policy applied in this work has a memory saving of the performance of the previous agents until the current state and is updated at every sampled learning step. It is based on the reward shaping mechanism introduced by Buhmann et al. (2011) [28]. This remembering makes it possible for the agent to know the actions that led to the best-designed molecules so far, which are the ones with the highest score. It speeds up the system’s learning. The agent’s remembering policy is presented in Equation (1) [29].

(1) $$J\left(\theta \right)=\frac{\left(1-\alpha \right)}{N}\;\underset{m\epsilon M}{\sum }J_{mol}\left(A,\mathbb{P},\;A_{m};\theta \right)+\frac{\alpha }{N}\;\underset{\tilde{m}\epsilon \tilde{M}}{\sum }J_{mol}\left(A,\tilde{A},\;\tilde{A_{m}};\theta \right)$$

where *J*(*θ*) is the agent’s remember policy; *α* is a scaling factor based on the contribution of the best agent; *N* is the number of molecules sampled; *M* is the set of molecules, *m*, generated in the current agent’s epoch; $J_{mol}\;$is the policy for each molecule; A is the current agent; ℙ is the previous model; $A_{m}$ is the set of actions chosen to build a molecule in the current agent’s epoch; the “~” means that it is the best so far.

The molecule’s remember policy is presented in Equation (2) [29].

(2) $$J_{mol}\left(\theta \right)={\left[\log P{\left(B\right)}_{B}-\left(logP{\left(B\right)}_{Ref.}+\sigma S\left(B\right)\right)\right]}^{2}$$

where *σ* is a scaling factor treated as a hyperparameter, which tunes the contribution of the score; $P{\left(B\right)}_{B}\;$is the probability of choosing the sequence of actions B in the model B; $P{\left(B\right)}_{Ref.\;}$ is the probability of the reference model for the same sequence of actions; $S\left(B\right)\;$is the score for the molecule generated by the actions B.

**Figure 4:**
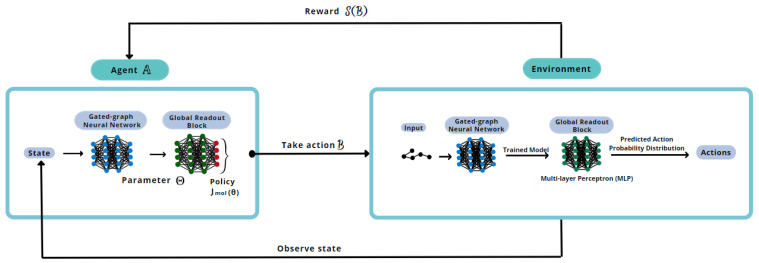
Reinforcement learning model’s training scheme.

To better understand the proposed methodology, a reinforcement learning training scheme is illustrated in Figure 4.

The deep reinforcement learning will go through this loop until the defined number of epochs is completed. Every batch of reinforcement implies a new training of the generation model, utilizing the best model trained without reinforcement. The system’s output, namely generative and reinforcement learning, is the best set of the requested number of new molecules to be designed. 

## 3. Results and Discussion

The architecture of the deep reinforcement learning used throughout this work is presented in Table 1. Due to the nature of the models, these hyperparameters were defined following a sensitivity analysis approach. 

The reinforcement learning epochs are subsequent to the generative ones in numeration. The generative neural network was trained for 1000 epochs. Meanwhile, the reinforcement learning one was trained for 500 epochs. The training was performed in a Linux server environment through a VirtualBox installed in a Windows 10 system. The server has an AMD Ryzen 9 5900X 12-Core Processor 3.79 GHz; 32.0 GB of installed RAM; an operative system of 64 bits; and an NVIDIA GeForce RTX 3060 GPU. The Oracle VM VirtualBox has an Ubuntu 64-bit operative system. 

Epoch 1040 was chosen as the agent model given its performance in the evaluation step. The evaluation results for the selected epoch are presented in Table 2, while the evaluation metrics are presented in Table 3. 

It is possible to visualize in Table 2 the percentage of valid molecules (PV); the percentage of molecules that were finished through the sampling of finish action (PPT); and the percentage of valid molecules in the set of PPT molecules (PVPT). These all are 1, which means that 100% of the molecules designed from this training epoch were valid and finished. On the other hand, the percentage of unique molecules in the set (PU) was 0.9, which means that 90% of the designed molecules in that training epoch were unique, and 10% of those were repeated. Those four metrics are especially important and must be analyzed when choosing the best epoch. Furthermore, they are essential to understand the choices made and their impact during training. Moreover, the average number of nodes per graph in the set ($\nu_{av}$) and the average number of edges per node (${ɛ}_{av}$) are relevant and must be considered when trying to understand if the designed molecules have a chemical structure like the database’s molecules. The uniformity-completeness Jensen–Shannon divergence (UC-JSD) is a metric used to choose the best action to take on the generative model based on the minimization of this value.

**Figure 5:**
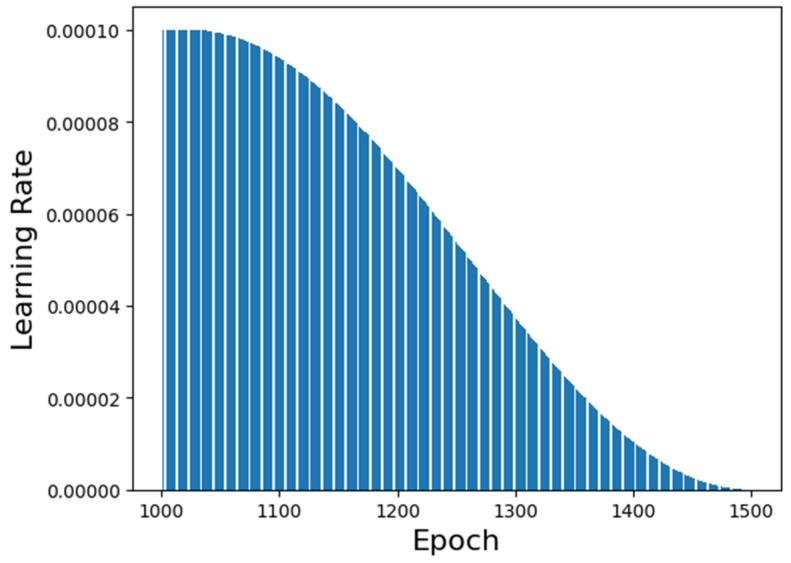
Reinforcement learning’s learning rate.

The learning rate performance along the training is shown in Figure 5. It is possible to visualize that the chosen generation epoch has a high learning rate value but not the highest. This fact is relevant to determine if the neural network’s prediction is biased or overfitted. The lower the learning rate’s value, the more overfitted the neural network is. Similarly, the higher the learning rate’s value, the more biased the neural network is.

**Figure 6:**
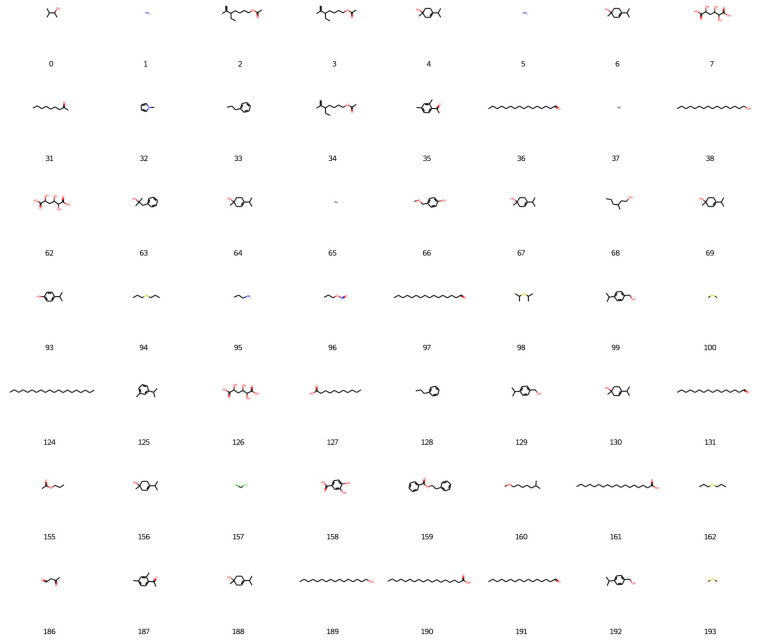
New designed molecules from deep reinforcement learning part 1.

**Figure 7:**
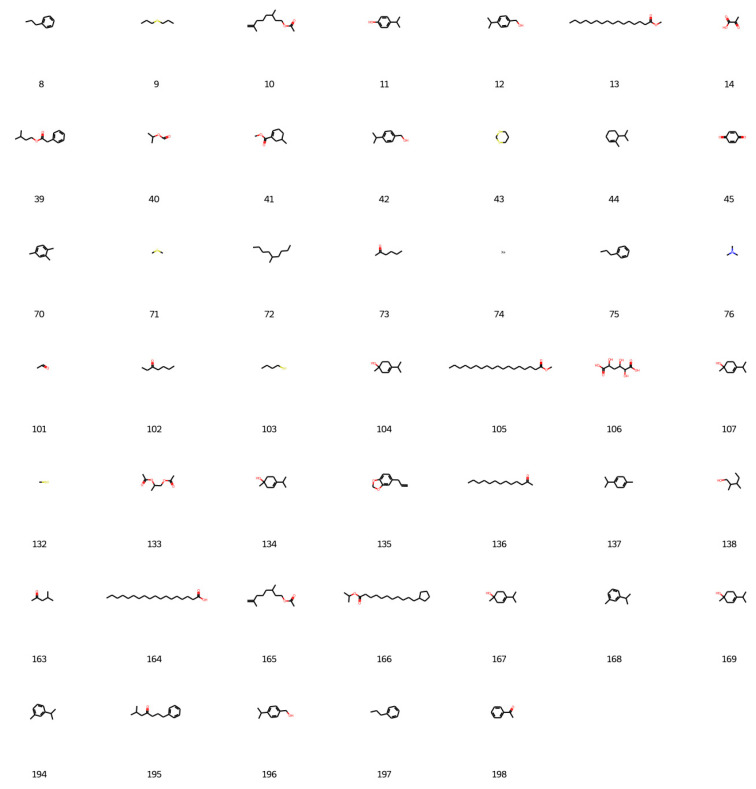
New designed molecules from deep reinforcement learning part 2.

**Figure 8:**
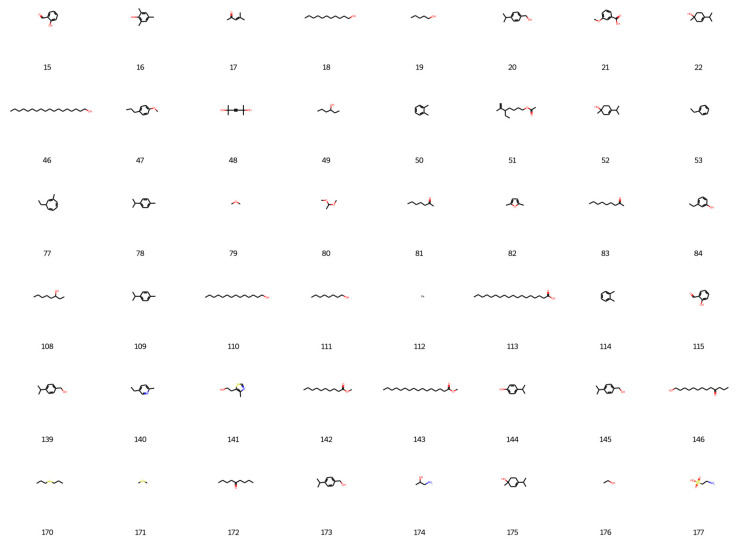
New designed molecules from deep reinforcement learning part 3.

**Figure 9:**
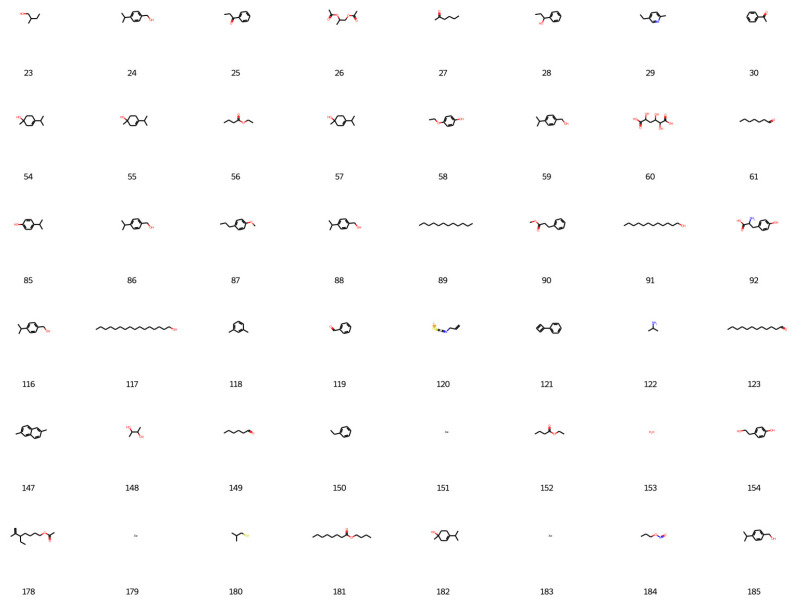
New designed molecules from deep reinforcement learning part 4.

Based on the best agent chosen, 200 molecules were generated, and 198 were considered valid by the network, as shown in Figure 6, Figure 7, Figure 8 and Figure 9. 

It is relevant to notice that the reinforcement learning training took approximately 6 h and 18 min, while the deep generative model took about 7 days, 15 h, and 31 min. Although the generative model epochs were double the reinforcement learning ones, the discrepancy in the amount of time required to train each SciML technique is prominent.

As previously explained, during the reinforcement learning training, the molecules received a score based on the rewards metrics: SAScore, NPScore, and target size. The metrics SAScore and NPScore are the main results of this section; it is of note that they are the target metrics of the work. The SAScore ranges from 1 to 10, with the lowest value meaning the easiest to synthesize and the highest one the hardest. The NPScore goes from −5 to 5. The higher the value, the more similar the newly designed molecule is to a natural product. Based on the work of Chen et al. (2019) [26], a score higher than zero can be classified as a natural product. The pseudo-natural values are around zero on the scale with positive and negative values. Within this frame, values of SAScore below three and NPScore higher than zero will be considered optimal for the goal presented in this work: to design molecules that are easy to be synthesized and that can be considered as natural.

**Figure 10:**
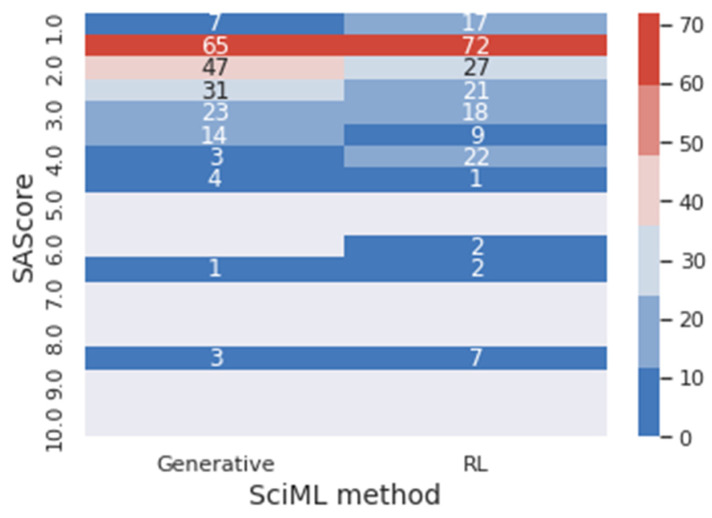
Heatmap of the frequency of values in the range of the SAScore for the generative and the reinforcement learning model.

**Figure 11:**
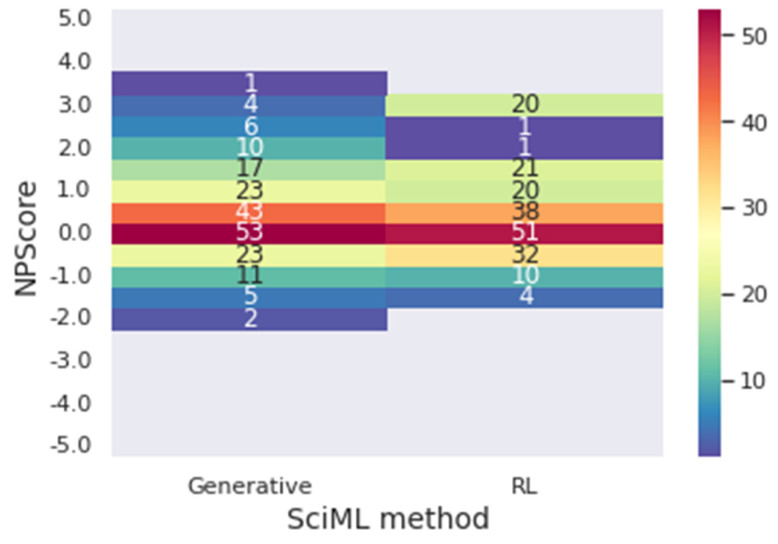
Heatmap of the frequency of values in the range of the NPScore for the generative and the reinforcement learning model.

To verify the impact of the reinforcement learning method on the results, the reward metrics were calculated for the molecules generated through the deep generative model in the last section. Figure 10 presents a heat map of the frequency of the SAScore range for generative and reinforcement learning (RL) results. In the same context, Figure 11 presents a heat map for the NPScore range. 

In Figure 10, it is possible to visualize that 81.31% of the molecules generated through the generative method obtained optimal values for the SAScore. In comparison, 79.80% of the molecules obtained through reinforcement learning achieved this result. However, 57.58% of the reinforcement learning’s molecules are between 1 and 2 for the SAScore, while only 47.97% of the generative method’s molecules are in this range. This means that even though the generative model can design molecules that present good results in the target metric, the reinforcement learning designed molecules with better performance.

In Figure 11, it is possible to conclude that, once again, the reinforcement learning designs molecules with better performance regarding the target metrics. The generative model presented 72.21% of the molecules in the optimal range of results for the NPScore. However, 65.74% of those are between 0 and 1, in the pseudo-natural range. At the same time, 74.75% of the reinforcement learning molecules have optimal results, and only 58.80% are in the pseudo-natural range. In achieving optimal results for both target metrics simultaneously, 53.53% of the molecules achieved the optimal metrics for the generative, and 55.55% achieved it for reinforcement learning, demonstrating that using reinforcement learning improves the results when both metrics are considered. Furthermore, when reinforcement learning is used, there is a significantly higher number of molecules with the best values of the metrics. For instance, there are 20 molecules with NPscore of 3 against the 5 molecules provided by the solely generative approach.

An assessment was performed to evaluate the 200 molecules designed. This assessment was based on the following parameters: number of molecules that are valid/invalid; existent/non-existent; already used in the flavor industry and are/are not in the FlavorDB website database; are not yet used in the flavor industry and are/are not in the FlavorDB website database. The evaluation is presented in Table 4. For this purpose, the FlavorDB website was used as reference since the database used throughout this work was scraped from this website, alongside the PubChem [30]. It is important to notice that the database that was scraped for this work from the FlavorDB website is only a small part of the website database. Therefore, the designed molecules used in the flavor industry and in the FlavorDB website are not necessarily part of the database used to train the model. 

As previously discussed, the reinforcement learning model designed 198 valid molecules and two invalid molecules. Considering the 198 valid molecules, 6 of them, even though they are considered valid and have canonical SMILES, are not recognized by the PubChem [30] online database, representing 3% of the designed molecules. Two of the non-existent molecules SMILES are presented in Equations (3) and (4). The application of those molecules in the flavor industry requires the development of a reaction path to synthetize them.

(3) $$C=C\left(C\right)C\left(\mathrm{CC}\right)\mathrm{CCCCOC}\left(C\right)=O$$

(4) $$\mathrm{CC}\left(C\right)\mathrm{OC}\left(=O\right)\mathrm{CCCCCCCCCCC}1\mathrm{CCCC}1$$

One valuable aspect of the assessment of the result is the establishment of the proposed methodology’s goals. The generative model is constructed to design molecules to be implemented in flavor engineering products and developments. In this work, it is implemented as the environment of a reinforcement learning model. It does not mean that all the designed molecules will not have a defined reaction path or that all the designed molecules are not yet applied in the flavor industry. The proposal of this methodology aims to be a resourceful tool for flavor-based products’ development. For this instance, the output must also contain options of molecules already used in the flavor industry and not yet used in the flavor industry but are common in the market and available for research. It is important to highlight that 99% of the designed molecules are valid, 96% already exist and have defined reaction paths, and 63.5% can be found in the literature as having already been applied to the flavor industry.

Considering a more detailed assessment of the result regarding the presence of the molecules in the FlavorDB website database [20] or the available information on the PubChem website [30], the following was found:

1. Overall, 50.5% are used in the flavor industry and are in the FlavorDB website database with a corresponding flavor descriptor. These molecules also have a section entitled “Food Additives and Ingredients” that specifies the regulation and applicability associated with the molecules;
2. Another 13% are used in the flavor industry. However, they are not in the FlavorDB website database. On the PubChem website, those molecules have a section entitled “Food Additives and Ingredients” that specifies the regulation and applicability associated with the molecules. All the molecules considered in this category are regulated by the European Union and/or the FDA in the United States of America;
3. Another 13% are not used yet in the flavor industry even though they are in the FlavorDB website database. However, these molecules do not have a corresponding flavor descriptor, and it is impossible to find in the literature its utilization on the flavor industry;
4. Overall, 19.5% are not used yet in the flavor industry and are not in the FlavorDB website database. Moreover, on the PubChem website, those molecules are associated with other industrial applications and do not have the section “Food Additives and Ingredients”.

The total percentage of designed molecules not yet employed in the flavor industry is 32.5%. This percentage corresponds to molecules applied to the pharmaceutical industry, package production, lubricants, solvents, other flavors’ precursor, or perfumes. As an example, two of the obtained molecules in this 32.5% will be further assessed in the following paragraphs. 

**Figure 12:**
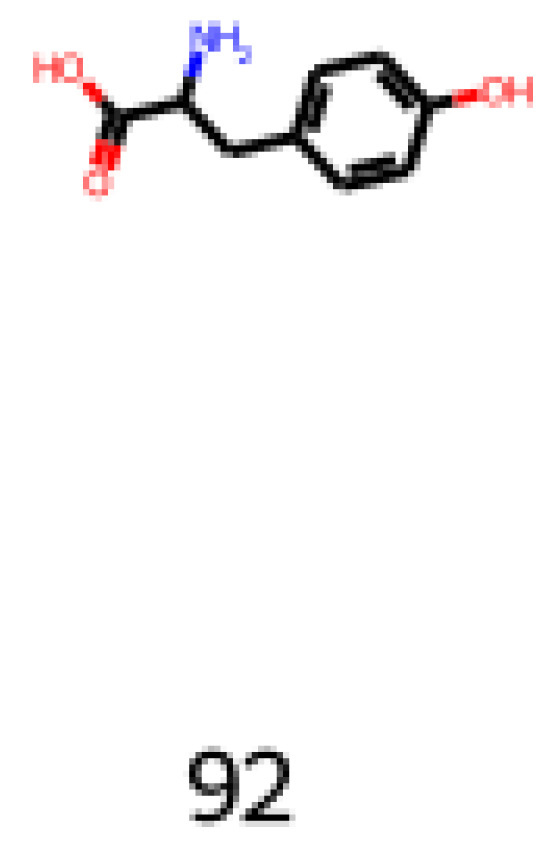
Image obtained as an output of the reinforcement learning of the 2-amino-3-(4-hydroxyphenyl)propanoic acid.

One of the molecules to be assessed is 2-amino-3-(4-hydroxyphenyl)propanoic acid (CAS number: 556-03-6), also known as DL-Tyrosine, shown in Equation (5) and Figure 12. It is an aromatic nonessential amino acid used in the pharmaceutical industry and biochemistry applications. It is a precursor for numerous neurotransmitters, for example, dopamine. It is an interesting molecule to be analyzed and considered for flavor-based products, keeping in mind the pharmacological properties.

(5) $$\mathrm{NC}\left(\mathrm{Cc}1\mathrm{ccc}\left(O\right)\mathrm{cc}1\right)C\left(=O\right)O$$

**Figure 13:**
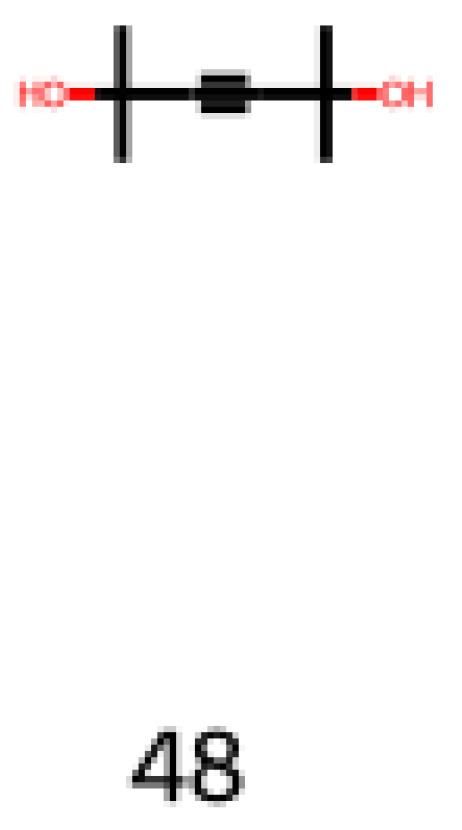
Image obtained as an output of the reinforcement learning of the 2,5-dimethylhex-3-yne-2,5-diol.

The other molecule to be assessed is 2,5-dimethylhex-3-yne-2,5-diol (CAS number: 142-30-3), shown in Equation (6) and Figure 13. It is a versatile molecule with numerous applications in the industry, such as use in antifoaming agents, paint components, coupling agents in resin coatings, wire-drawing lubricants, peroxide catalysts, and fragrance compounds. It is an interesting molecule to consider regarding its versatility, and it is already applied as a fragrance compound. However, its toxicology must be assessed for ingestion to be applied in the flavor industry.

(6) $$\mathrm{CC}\left(C\right)\left(C\#\mathrm{CC}\left(C\right)\left(C\right)O\right)O$$

Two molecules will be further analyzed in the following paragraphs to exemplify the molecules obtained from the reinforcement learning model output, which are in the 69.5% of the molecules already used in the flavor industry.

**Figure 14:**
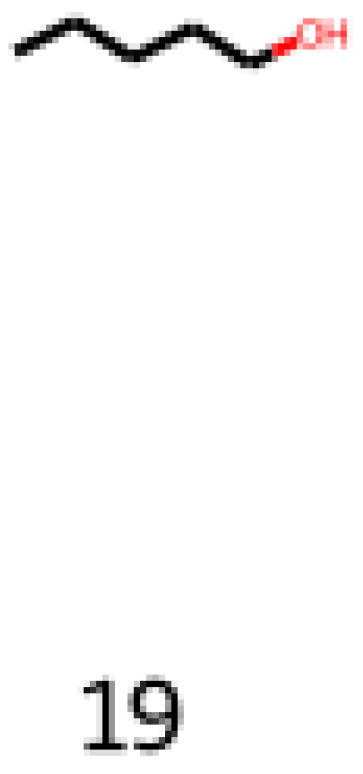
Image obtained as an output of the reinforcement learning of the pentan-1-ol.

The first assessed molecule of this category is pentan-1-ol (CAS number: 71-41-0), shown in Equation (7) and Figure 14. It is a molecule with an alcohol functional group and has associated flavor profiles in the balsamic, fruit, oil, fusel, sweet, vanilla, balsam, green, pungent, and yeast descriptors.

(7) CCCCCO

**Figure 15:**
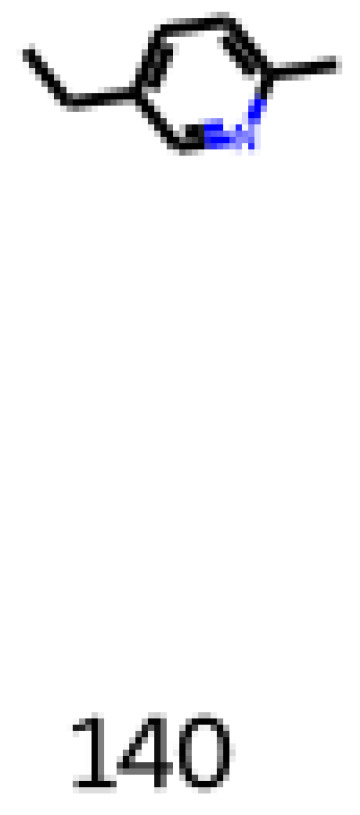
Image obtained as an output of the reinforcement learning of the 5-ethyl-2-methylpyridine.

The second one is 5-ethyl-2-methylpyridine (CAS number: 104-90-5), shown in Equation (8) and Figure 15. It is an aromatic molecule and has associated flavor profiles the raw, savory, roasted, potato, earthy, strong, and nutty descriptors.

(8) $$\mathrm{CCc}1\mathrm{ccc}\left(C\right)\mathrm{nc}1$$

Through the assessment of the results, it is possible to conclude that the reinforcement learning model, alongside the generative model, can design molecules already used in the flavor industry or in other industry sections, with better results than the generative model itself. It was also possible to visualize that this procedure to develop flavor-based products does not require the development of new reaction paths for new molecules. 

In fact, it establishes that it is possible to find solutions for the flavor industry with molecules already available in the market. This is a helpful result, as it significantly facilitates the development processes. Furthermore, it simplifies the regulation step, as one can obtain molecules already regulated in the food market. Overall, the methodology provides new insights for novel applications of molecules already available in the food industry.

## 4. Conclusions

The proposed work raises a new perspective in flavor engineering based on scientific machine learning. The combination of a generative and a reinforcement learning models was proposed to generate new flavor molecules. The focus was to generate flavoring molecules that could be synthesized and applied in the natural products industry, proposing a tool to find molecules that are already available in the market and can be applied, or not, in the flavor industry and new molecules that require the development of a reaction path and can be studied and analyzed to be used in flavor-based products. Therefore, this research addresses an emerging challenge present in the flavor engineering industry.

It was demonstrated that employing the proposed deep reinforcement learning method led to better results regarding the synthetic accessibility score (SAScore) and the natural product-likeness score (NPScore). It obtained 10% more molecules within the optimal results than the deep generative model. The designed molecules obtained as an output of the reinforcement learning model’s generation were assessed regarding their existence or not in the market and whether they are already used in the flavor industry or not. A further analysis was performed regarding the information available on websites such as FlavorDB and PubChem.

Hence, a new novel framework was created to enhance product engineering developments, providing an innovative approach to generating molecules with specific desired characteristics and chemical properties.

## Figures and Tables

**Table 1:** Deep reinforcement learning hyperparameters.

Parameters	Deep Reinforcement Learning’s Value
A	0.50
Batch size	20
Block size	1000
Epochs	500
Generation epoch	1040
GGNN activation function	SELU
GGNN depth	4
GGNN dropout probability	0
GGNN hidden dimension	250
GGNN width	100
Initial learning rate	1.00 × 10^−4^
Learning rate decay factor	0.99
Learning rate decay interval	10
Loss function	Kullback–Leibler divergence
Maximum relative learning rate	1.00
Message passing layers	3
Message size–input size of GRU	100
Minimum relative learning rate	1.00 × 10^−4^
MLP activation function	SoftMax
MLP depth(Layers 1 and 2)	4
MLP dropout probability (Layers 1 and 2)	0
MLP hidden dimension (Layers 1 and 2)	500
Number of samples	200
Optimizer	Adam
σ	20
Weight decay	0
Weight initialization	Uniform

**Table 2:** Evaluation results for the agent model chosen.

Epoch	PV (0–1)	PVPT (0–1)	PPT (0–1)	$\nu_{av}$	${ɛ}_{av}$	PU (0–1)
1040	1.00	1.00	1.00	9.35	1.88	0.90

**Table 3:** Evaluation metrics.

Metrics	Description
PV	Percentage of valid molecules in the set
PU	Percentage of unique molecules in the set
PPT	Percentage of molecules that were finished through sampling of finish action
PVPT	Percentage of valid molecules in the set of PPT molecules
$\nu_{av}$	Average number of nodes per graph in the set
${ɛ}_{av}$	Average number of edges per node
UC-JSD	Uniformity-completeness Jensen–Shannon divergence

**Table 4:** Designed molecules assessment results.

Categories	Number of Molecules	Percentage of Molecules (%)
Valid molecules	198	99
Invalid molecules	2	1
Existent	192	96
Non-existent	6	3
Used in the flavor industry	127	63.5
Not yet used in the flavor industry	65	32.5
Used in the flavor industry and are in the FlavorDB website database	101	50.5
Used in the flavor industry and are not in the FlavorDB website database	26	13
Not yet used in the flavor industry and are in the FlavorDB website database	26	13
Not yet used in the flavor industry and are not in the FlavorDB website database	39	19.5

## Data Availability

Publicly available datasets were analyzed in this study. This data can be found here: http://cosylab.iiitd.edu.in/flavordb (accessed on 5 April 2022).

## References

[1] The Editors of Encyclopaedia Britannica Flavour. https://www.britannica.com/topic/flavor.

[2] Reineccius G. (2006). Flavor Chemistry and Technology.

[3] Fortune Business Insights (2022). Food Flavors Market Size, Share & COVID-19 Impact Analysis, By Type (Natural and Synthetic), by Application (Bakery, Beverages, Confectionery, Dairy, Convenience Food, Snacks, and Others), and Regional Forecast, 2021–2028.

[4] Sumesh Kumar R.D. (2021). Food Flavors Market by Type (Natural, and Artificial), and End-User (Beverages, Dairy & Frozen Products, Bakery & Confectionery, Savory & Snacks, Animal & Pet Food): Global Opportunity Analysis and Industry Forecast, 2021–2030.

[5] (2021). Flavors & Fragrances Market by Ingredients (Natural, Synthetic), End use (Beverage, Savory & Snacks, Bakery, Dairy Products, Confectionery, Consumer Products, Fine Fragrances), and Region (Asia Pacific, North America, Europe)—Global Forecast to 2026.

[6] (2021). Regulation (EC) No 1334/2008 of the European Parliament and of the Council of 16 December 2008 on Flavourings and Certain Food Ingredients with Flavouring Properties for Use in and on Foods and Amending Council Regulation (EEC) No 1601/91, Regulat. http://data.europa.eu/eli/reg/2008/1334/oj.

[7] Bi K., Zhang D., Qiu T., Huang Y. (2020). GC-MS fingerprints profiling using machine learning models for food flavor prediction. Processes.

[8] François-lavet V., Henderson P., Islam R., Bellemare M.G., François-lavet V., Pineau J., Bellemare M.G. (2018). An Introduction to Deep Reinforcement Learning. arXiv.

[9] Yeh J.F., Su P.H., Huang S.H., Chiang T.C. Snake game AI: Movement rating functions and evolutionary algorithm-based optimization. Proceedings of the 2016 Conference on Technologies and Applications of Artificial Intelligence (TAAI).

[10] Mnih V., Kavukcuoglu K., Silver D., Rusu A.A., Veness J., Bellemare M.G., Graves A., Riedmiller M., Fidjeland A.K., Ostrovski G. (2015). Human-level control through deep reinforcement learning. Nature.

[11] Leong Y.X., Lee Y.H., Koh C.S.L., Phan-Quang G.C., Han X., Phang I.Y., Ling X.Y. (2021). Surface-Enhanced Raman Scattering (SERS) Taster: A Machine-Learning-Driven Multireceptor Platform for Multiplex Profiling of Wine Flavors. Nano Lett..

[12] Mercado R., Rastemo T., Lindelof E., Klambauer G., Engkvist O., Chen H., Bjerrum E.J. (2021). Graph networks for molecular design. Mach. Learn. Sci. Technol..

[13] Zhou J., Cui G., Hu S., Zhang Z., Yang C., Liu Z., Wang L., Li C., Sun M. (2020). Graph Neural Networks: A Review of Methods and Applications. AI Open.

[14] Mousavi S.S., Schukat M., Howley E. (2018). Deep Reinforcement Learning: An Overview. Lect. Notes Networks Syst..

[15] Shao K., Tang Z., Zhu Y., Li N., Zhao D. (2019). A Survey of Deep Reinforcement Learning in Video Games. arXiv.

[16] Olivecrona M., Blaschke T., Engkvist O., Chen H. (2017). Molecular de-novo design through deep reinforcement learning. J. Cheminform..

[17] Born J., Manica M., Oskooei A., Cadow J., Markert G., Rodríguez Martínez M. (2021). PaccMannRL: De novo generation of hit-like anticancer molecules from transcriptomic data via reinforcement learning. iScience.

[18] Queiroz L.P., Rebello C.M., Costa E.A., Santana V.V., Rodrigues B.C.L., Rodrigues A.E., Ribeiro A.M., Nogueira I.B.R. (2022). Generating Flavors Using Scientific Machine Learning.

[19] Zhou Z., Kearnes S., Li L., Zare R.N., Riley P. (2019). Optimization of Molecules via Deep Reinforcement Learning. Sci. Rep..

[20] Bagler G. FlavorDB. https://cosylab.iiitd.edu.in/flavordb/.

[21] Li Y., Zemel R., Brockschmidt M., Tarlow D. Gated graph sequence neural networks. Proceedings of the 4th International Conference on Learning Representations, ICLR 2016.

[22] Pereira T., Abbasi M., Ribeiro B., Arrais J.P. (2021). Diversity oriented Deep Reinforcement Learning for targeted molecule generation. J. Cheminform..

[23] Nwankpa C., Ijomah W., Gachagan A., Marshall S. (2018). Activation Functions: Comparison of trends in Practice and Research for Deep Learning. arXiv.

[24] Ertl P., Schuffenhauer A. (2009). Estimation of synthetic accessibility score of drug-like molecules based on molecular complexity and fragment contributions. J. Cheminform..

[25] Ertl P., Roggo S., Schuffenhauer A. (2008). Natural product-likeness score and its application for prioritization of compound libraries. J. Chem. Inf. Model..

[26] Chen Y., Stork C., Hirte S., Kirchmair J. (2019). NP-scout: Machine learning approach for the quantification and visualization of the natural product-likeness of small molecules. Biomolecules.

[27] Karageorgis G., Foley D.J., Laraia L., Waldmann H. (2020). Principle and design of pseudo-natural products. Nat. Chem..

[28] Buhmann M.D. (2000). Buhmann Radial Basis Functions. Acta Numer..

[29] Atance S.R., Diez J.V., Engkvist O., Olsson S., Mercado R. (2021). De novo drug design using reinforcement learning with graph-based deep generative models. ChemRxiv.

[30] Kim S., Chen J., Cheng T., Gindulyte A., He J., He S., Li Q., Shoemaker B.A., Thiessen P.A., Yu B. (2021). PubChem in 2021: New data content and improved web interfaces. Nucleic Acids Res..

